# 4-[(2,4-Dimethyl-1,3-oxazol-5-yl)meth­yl]-4-hydr­oxy-2-methyl­isoquinoline-1,3(2*H*,4*H*)-dione

**DOI:** 10.1107/S1600536810007397

**Published:** 2010-03-03

**Authors:** Hoong-Kun Fun, Jia Hao Goh, Haitao Yu, Yan Zhang

**Affiliations:** a X-ray Crystallography Unit, School of Physics, Universiti Sains Malaysia, 11800 USM, Penang, Malaysia; b School of Chemistry and Chemical Engineering, Nanjing University, Nanjing 210093, People’s Republic of China

## Abstract

In the title isoquinolinedione derivative, C_16_H_16_N_2_O_4_, the piperidine ring in the tetra­hydro­isoquinoline unit adopts a half-boat conformation. The essentially planar oxazole ring [maximum deviation = 0.004 (2) Å] is inclined at a dihedral angle of 36.00 (8)° to the tetra­hydro­isoquinoline unit. In the crystal structure, pairs of inter­molecular C—H⋯O and O—H⋯N inter­actions link the mol­ecules into chains incorporating *R*_2_^2^(9) ring motifs. Two neighbouring chains are further inter­connected by inter­molecular C—H⋯O inter­actions into chains two mol­ecules wide along the *a* axis.

## Related literature

For general background to and applications of the title isoquinoline compound, see: Chen *et al.* (2006 ▶); Hall *et al.* (1994 ▶); Malamas & Hohman (1994 ▶); Mitchell *et al.* (1995 ▶, 2000 ▶). For ring conformations, see: Cremer & Pople (1975 ▶). For hydrogen-bond motifs, see: Bernstein *et al.* (1995 ▶). For related structures, see: Subbiah Pandi *et al.* (2002 ▶); Wang *et al.* (2000 ▶). For bond-length data, see: Allen *et al.* (1987 ▶). For the stability of the temperature controller used for the data collection, see: Cosier & Glazer (1986 ▶).
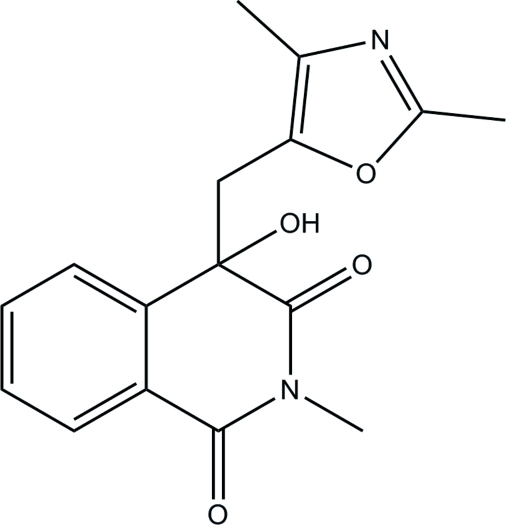


## Experimental

### 

#### Crystal data

C_16_H_16_N_2_O_4_
*M* *_r_* = 300.31
Triclinic, 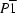

*a* = 8.3866 (5) Å
*b* = 8.8044 (5) Å
*c* = 10.6734 (7) Å
α = 103.997 (3)°
β = 90.025 (3)°
γ = 112.663 (2)°
*V* = 701.80 (7) Å^3^
*Z* = 2
Mo *K*α radiation
μ = 0.10 mm^−1^
*T* = 100 K
0.24 × 0.19 × 0.08 mm

#### Data collection

Bruker SMART APEXII CCD area-detector diffractometer
Absorption correction: multi-scan (*SADABS*; Bruker, 2009 ▶) *T*_min_ = 0.976, *T*_max_ = 0.992
6623 measured reflections
3198 independent reflections
2401 reflections with *I* > 2σ(*I*)
*R*_int_ = 0.034

#### Refinement

*R*[*F* ^2^ > 2σ(*F* ^2^)] = 0.050
*wR*(*F* ^2^) = 0.135
*S* = 1.04
3198 reflections
263 parameters
All H-atom parameters refined
Δρ_max_ = 0.40 e Å^−3^
Δρ_min_ = −0.28 e Å^−3^

### 

Data collection: *APEX2* (Bruker, 2009 ▶); cell refinement: *SAINT* (Bruker, 2009 ▶); data reduction: *SAINT*; program(s) used to solve structure: *SHELXTL* (Sheldrick, 2008 ▶); program(s) used to refine structure: *SHELXTL*; molecular graphics: *SHELXTL*; software used to prepare material for publication: *SHELXTL* and *PLATON* (Spek, 2009 ▶).

## Supplementary Material

Crystal structure: contains datablocks global, I. DOI: 10.1107/S1600536810007397/sj2737sup1.cif

Structure factors: contains datablocks I. DOI: 10.1107/S1600536810007397/sj2737Isup2.hkl

Additional supplementary materials:  crystallographic information; 3D view; checkCIF report

## Figures and Tables

**Table 1:** Hydrogen-bond geometry (Å, °)

*D*—H⋯*A*	*D*—H	H⋯*A*	*D*⋯*A*	*D*—H⋯*A*
O3—H1*O*3⋯N2^i^	0.87 (3)	2.04 (3)	2.847 (2)	153 (3)
C16—H16*A*⋯O1^ii^	0.96 (3)	2.28 (3)	3.162 (2)	153 (2)
C16—H16*B*⋯O1^iii^	1.01 (3)	2.50 (3)	3.270 (2)	132.9 (19)

Symmetry codes: (i) 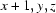; (ii) 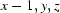; (iii) 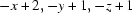.

**Fig. 1.**
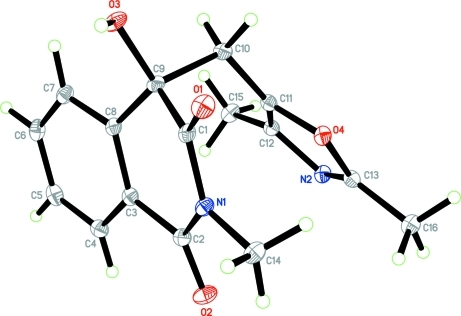
The structure of the title compound, showing the atom numbering scheme. Displacement ellipsoids are drawn at the 30% probability level.

**Fig. 2.**
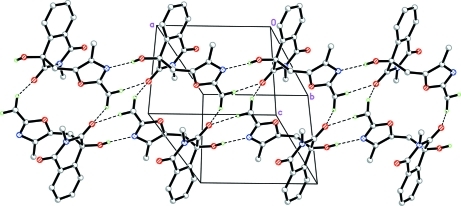
The crystal structure of the title compound, showing two-molecule-wide chain along the a axis. H atoms not involved in intermolecular interactions (dashed lines) have been omitted for clarity.

## supplementary crystallographic information

### Comment

A series of isoquinoline-1,3,4-trione derivatives were identified as novel and potent inhibitors of caspase-3 through structural modification of the original compounds from high-throughput screening (Chen *et al.*, 2006). Moreover, the series of isoquinoline-1,3,4-triones were found to be fast-acting post-emergence herbicides, producing symptoms of desiccation (Mitchell *et al.*, 2000). These redox-active compounds are very potent stimulators of the light-dependent consumption of oxygen at photosystem in isolated chloroplasts (Mitchell *et al.*, 1995). Isoquinoline-1,3,4-trione derivatives have a variety of biological activities and are synthetic precursors for many naturally occuring alkaloids (Hall *et al.*, 1994; Malamas & Hohman, 1994). The crystal structure of the related *Z*-2-methyl-3'-phenyl-spiro[isoquinoline-4,2'-oxirane]-1,3-dione has been reported (Wang *et al.*, 2000).

In the title isoquinoline-1,3-dione compound (Fig. 1), the piperidine ring (C1/N1/C2/C3/C8/C9) in the 1,2,3,4-tetrahydroisoquinolin moiety adopts a half-boat conformation (Cremer & Pople, 1975) with puckering parameters of Q = 0.3114 (19) Å, θ = 71.4 (3)° and φ = 114.9 (4)°. The oxazole ring (C11/C12/N2/C13/O4) is essentially planar with maximum deviation of -0.004 (2) Å at atom C13. The oxazole ring is inclined at a dihedral angle of 36.00 (8)° with the mean plane through 1,2,3,4-tetrahydroisoquinolin moiety. Bond lengths (Allen *et al.*, 1987) and angles are normal and comparable to those related isoquinoline-1,3-dione structures (Wang *et al.*, 2000; Subbiah Pandi *et al.*, 2002).

In the crystal structure (Fig. 2), intermolecular O3—H1O3···N2 and C16—H16A···O1 hydrogen bonds (Table 1) link the molecules into one-dimensional chains along *a* axis incorporating *R*^2^_2_(9) ring motifs (Bernstein *et al.*, 1995). Two neighbouring chains are further interconnected by intermolecular C16—H16B···O1 hydrogen bonds into two-molecule-wide chains along the same axis.

### Experimental

The title compound was obtained in the reaction between 1,3,4(2*H*)-isoquinolinetrione and 2,4,5-trimethyloxazole. The compound was purified by flash column chromatography in ethyl acetate and petroleum ether. X-ray quality single crystals of the title compound were obtained from slow evaporation of a chloroform solution. *M.p.* 434–436 K.

### Refinement

All the H atoms were located from difference Fourier map [range of C—H = 0.91 (2) - 1.01 (3) Å] and allowed to refine freely.

### Crystal data

**Table:**

C_16_H_16_N_2_O_4_	*Z* = 2
*M**_r_* = 300.31	*F*(000) = 316
Triclinic, *P*1	*D*_x_ = 1.421 Mg m^−^^3^
Hall symbol: -P 1	Mo *K*α radiation, λ = 0.71073 Å
*a* = 8.3866 (5) Å	Cell parameters from 1833 reflections
*b* = 8.8044 (5) Å	θ = 4.4–32.7°
*c* = 10.6734 (7) Å	µ = 0.10 mm^−^^1^
α = 103.997 (3)°	*T* = 100 K
β = 90.025 (3)°	Block, colourless
γ = 112.663 (2)°	0.24 × 0.19 × 0.08 mm
*V* = 701.80 (7) Å^3^	

### Data collection

**Table:**

Bruker SMART APEXII CCD area-detector diffractometer	3198 independent reflections
Radiation source: fine-focus sealed tube	2401 reflections with *I* > 2σ(*I*)
graphite	*R*_int_ = 0.034
φ and ω scans	θ_max_ = 27.5°, θ_min_ = 2.7°
Absorption correction: multi-scan (*SADABS*; Bruker, 2009)	*h* = −10→10
*T*_min_ = 0.976, *T*_max_ = 0.992	*k* = −11→11
6623 measured reflections	*l* = −13→13

### Refinement

**Table:**

Refinement on *F*^2^	Primary atom site location: structure-invariant direct methods
Least-squares matrix: full	Secondary atom site location: difference Fourier map
*R*[*F*^2^ > 2σ(*F*^2^)] = 0.050	Hydrogen site location: inferred from neighbouring sites
*wR*(*F*^2^) = 0.135	All H-atom parameters refined
*S* = 1.04	*w* = 1/[σ^2^(*F*_o_^2^) + (0.0731*P*)^2^ + 0.0844*P*] where *P* = (*F*_o_^2^ + 2*F*_c_^2^)/3
3198 reflections	(Δ/σ)_max_ < 0.001
263 parameters	Δρ_max_ = 0.40 e Å^−^^3^
0 restraints	Δρ_min_ = −0.28 e Å^−^^3^

### Special details

**Table:**

Experimental. The crystal was placed in the cold stream of an Oxford Cryosystems Cobra open-flow nitrogen cryostat (Cosier & Glazer, 1986) operating at 100.0 (1)K.
Geometry. All esds (except the esd in the dihedral angle between two l.s. planes) are estimated using the full covariance matrix. The cell esds are taken into account individually in the estimation of esds in distances, angles and torsion angles; correlations between esds in cell parameters are only used when they are defined by crystal symmetry. An approximate (isotropic) treatment of cell esds is used for estimating esds involving l.s. planes.
Refinement. Refinement of F^2^ against ALL reflections. The weighted R-factor wR and goodness of fit S are based on F^2^, conventional R-factors R are based on F, with F set to zero for negative F^2^. The threshold expression of F^2^ > 2sigma(F^2^) is used only for calculating R-factors(gt) etc. and is not relevant to the choice of reflections for refinement. R-factors based on F^2^ are statistically about twice as large as those based on F, and R- factors based on ALL data will be even larger.

### Fractional atomic coordinates and isotropic or equivalent isotropic displacement parameters (Å^2^)

**Table:**

	*x*	*y*	*z*	*U*_iso_*/*U*_eq_	
O1	1.23014 (16)	0.40877 (16)	0.32581 (13)	0.0234 (3)	
O2	0.76462 (17)	0.27268 (17)	0.04028 (14)	0.0269 (3)	
O3	1.13932 (17)	0.07862 (17)	0.33591 (13)	0.0224 (3)	
O4	0.78937 (15)	0.36205 (15)	0.40053 (12)	0.0185 (3)	
N1	0.99911 (18)	0.34315 (18)	0.18332 (15)	0.0175 (3)	
N2	0.50707 (18)	0.22071 (19)	0.33559 (15)	0.0186 (3)	
C1	1.0928 (2)	0.3023 (2)	0.26656 (17)	0.0175 (4)	
C2	0.8503 (2)	0.2243 (2)	0.10092 (18)	0.0187 (4)	
C3	0.8116 (2)	0.0419 (2)	0.08846 (17)	0.0175 (4)	
C4	0.6900 (2)	−0.0817 (2)	−0.01212 (19)	0.0217 (4)	
C5	0.6559 (2)	−0.2517 (3)	−0.0275 (2)	0.0249 (4)	
C6	0.7436 (3)	−0.3003 (2)	0.0555 (2)	0.0249 (4)	
C7	0.8637 (2)	−0.1782 (2)	0.15593 (19)	0.0209 (4)	
C8	0.8972 (2)	−0.0063 (2)	0.17380 (17)	0.0171 (4)	
C9	1.0118 (2)	0.1260 (2)	0.29105 (18)	0.0175 (4)	
C10	0.8979 (2)	0.1419 (3)	0.40732 (18)	0.0191 (4)	
C11	0.7513 (2)	0.1891 (2)	0.38373 (17)	0.0172 (4)	
C12	0.5796 (2)	0.1033 (2)	0.34423 (17)	0.0180 (4)	
C13	0.6360 (2)	0.3692 (2)	0.36869 (17)	0.0179 (4)	
C14	1.0594 (3)	0.5246 (2)	0.1865 (2)	0.0238 (4)	
C15	0.4717 (3)	−0.0836 (2)	0.3101 (2)	0.0238 (4)	
C16	0.6387 (2)	0.5395 (2)	0.3724 (2)	0.0216 (4)	
H1O3	1.243 (4)	0.142 (3)	0.321 (3)	0.054 (8)*	
H4A	0.637 (3)	−0.043 (3)	−0.065 (2)	0.033 (6)*	
H5A	0.574 (3)	−0.336 (3)	−0.097 (2)	0.031 (6)*	
H6A	0.722 (3)	−0.420 (3)	0.045 (2)	0.029 (6)*	
H7A	0.924 (3)	−0.208 (2)	0.217 (2)	0.019 (5)*	
H10A	0.855 (3)	0.032 (3)	0.430 (2)	0.024 (5)*	
H10B	0.979 (3)	0.226 (3)	0.484 (2)	0.024 (5)*	
H14A	1.064 (3)	0.595 (3)	0.277 (3)	0.045 (7)*	
H14B	0.985 (3)	0.538 (3)	0.127 (3)	0.048 (7)*	
H14C	1.175 (4)	0.565 (3)	0.154 (3)	0.052 (8)*	
H15A	0.436 (3)	−0.124 (3)	0.215 (3)	0.037 (6)*	
H15B	0.530 (3)	−0.148 (3)	0.337 (2)	0.040 (7)*	
H15C	0.360 (3)	−0.114 (3)	0.349 (3)	0.047 (7)*	
H16A	0.526 (3)	0.529 (3)	0.344 (2)	0.037 (6)*	
H16B	0.682 (3)	0.622 (3)	0.461 (3)	0.039 (6)*	
H16C	0.718 (4)	0.594 (3)	0.314 (3)	0.053 (8)*	

### Atomic displacement parameters (Å^2^)

**Table:**

	*U*^11^	*U*^22^	*U*^33^	*U*^12^	*U*^13^	*U*^23^
O1	0.0134 (6)	0.0238 (7)	0.0300 (8)	0.0053 (5)	−0.0010 (5)	0.0053 (6)
O2	0.0215 (7)	0.0300 (8)	0.0333 (8)	0.0113 (6)	−0.0025 (6)	0.0136 (6)
O3	0.0105 (6)	0.0283 (7)	0.0340 (8)	0.0096 (6)	0.0033 (5)	0.0151 (6)
O4	0.0104 (6)	0.0223 (7)	0.0219 (7)	0.0072 (5)	0.0009 (5)	0.0026 (5)
N1	0.0133 (7)	0.0185 (7)	0.0224 (8)	0.0073 (6)	0.0023 (6)	0.0070 (6)
N2	0.0116 (7)	0.0221 (8)	0.0226 (8)	0.0077 (6)	0.0020 (6)	0.0052 (6)
C1	0.0132 (8)	0.0217 (9)	0.0203 (9)	0.0095 (7)	0.0049 (7)	0.0063 (7)
C2	0.0126 (8)	0.0260 (9)	0.0204 (9)	0.0092 (7)	0.0053 (7)	0.0088 (7)
C3	0.0113 (8)	0.0210 (9)	0.0203 (9)	0.0062 (7)	0.0045 (7)	0.0057 (7)
C4	0.0157 (9)	0.0290 (10)	0.0205 (9)	0.0086 (8)	0.0036 (7)	0.0073 (8)
C5	0.0167 (9)	0.0267 (10)	0.0240 (10)	0.0045 (8)	0.0035 (8)	0.0004 (8)
C6	0.0225 (10)	0.0201 (10)	0.0315 (11)	0.0087 (8)	0.0095 (8)	0.0055 (8)
C7	0.0167 (9)	0.0220 (9)	0.0271 (10)	0.0098 (8)	0.0055 (7)	0.0086 (8)
C8	0.0115 (8)	0.0212 (9)	0.0203 (9)	0.0075 (7)	0.0058 (7)	0.0070 (7)
C9	0.0112 (8)	0.0249 (9)	0.0210 (9)	0.0102 (7)	0.0026 (7)	0.0090 (7)
C10	0.0122 (8)	0.0270 (10)	0.0199 (9)	0.0086 (7)	0.0022 (7)	0.0079 (8)
C11	0.0133 (8)	0.0224 (9)	0.0162 (9)	0.0075 (7)	0.0033 (7)	0.0047 (7)
C12	0.0143 (8)	0.0236 (9)	0.0174 (9)	0.0087 (7)	0.0025 (7)	0.0055 (7)
C13	0.0108 (8)	0.0256 (10)	0.0169 (9)	0.0080 (7)	0.0009 (6)	0.0036 (7)
C14	0.0230 (10)	0.0190 (9)	0.0302 (11)	0.0084 (8)	0.0015 (8)	0.0077 (8)
C15	0.0157 (9)	0.0219 (10)	0.0333 (12)	0.0055 (8)	0.0027 (8)	0.0099 (8)
C16	0.0139 (9)	0.0216 (9)	0.0285 (11)	0.0067 (7)	0.0011 (8)	0.0059 (8)

### Geometric parameters (Å, °)

**Table:**

O1—C1	1.219 (2)	C6—H6A	0.98 (2)
O2—C2	1.219 (2)	C7—C8	1.392 (2)
O3—C9	1.4096 (19)	C7—H7A	0.97 (2)
O3—H1O3	0.87 (3)	C8—C9	1.510 (2)
O4—C13	1.3590 (19)	C9—C10	1.579 (3)
O4—C11	1.395 (2)	C10—C11	1.481 (2)
N1—C1	1.381 (2)	C10—H10A	0.99 (2)
N1—C2	1.405 (2)	C10—H10B	1.00 (2)
N1—C14	1.469 (2)	C11—C12	1.352 (2)
N2—C13	1.300 (2)	C12—C15	1.491 (3)
N2—C12	1.407 (2)	C13—C16	1.481 (3)
C1—C9	1.524 (2)	C14—H14A	1.01 (3)
C2—C3	1.482 (2)	C14—H14B	0.94 (3)
C3—C8	1.395 (2)	C14—H14C	0.99 (3)
C3—C4	1.398 (3)	C15—H15A	1.00 (3)
C4—C5	1.379 (3)	C15—H15B	0.97 (2)
C4—H4A	0.91 (2)	C15—H15C	0.99 (3)
C5—C6	1.391 (3)	C16—H16A	0.95 (2)
C5—H5A	0.96 (2)	C16—H16B	1.01 (3)
C6—C7	1.388 (3)	C16—H16C	0.98 (3)
			
C9—O3—H1O3	111.7 (18)	C1—C9—C10	106.39 (14)
C13—O4—C11	105.09 (13)	C11—C10—C9	115.98 (15)
C1—N1—C2	124.28 (14)	C11—C10—H10A	110.1 (12)
C1—N1—C14	116.33 (15)	C9—C10—H10A	106.9 (13)
C2—N1—C14	119.36 (14)	C11—C10—H10B	111.0 (12)
C13—N2—C12	105.17 (14)	C9—C10—H10B	106.8 (12)
O1—C1—N1	120.67 (16)	H10A—C10—H10B	105.3 (17)
O1—C1—C9	121.09 (15)	C12—C11—O4	107.30 (14)
N1—C1—C9	118.01 (15)	C12—C11—C10	135.61 (17)
O2—C2—N1	120.21 (16)	O4—C11—C10	117.05 (15)
O2—C2—C3	123.36 (17)	C11—C12—N2	108.95 (15)
N1—C2—C3	116.36 (14)	C11—C12—C15	129.76 (17)
C8—C3—C4	120.28 (16)	N2—C12—C15	121.29 (15)
C8—C3—C2	120.80 (16)	N2—C13—O4	113.49 (15)
C4—C3—C2	118.91 (16)	N2—C13—C16	129.41 (16)
C5—C4—C3	119.75 (18)	O4—C13—C16	117.08 (15)
C5—C4—H4A	123.9 (14)	N1—C14—H14A	110.5 (14)
C3—C4—H4A	116.4 (14)	N1—C14—H14B	109.2 (15)
C4—C5—C6	120.24 (18)	H14A—C14—H14B	112 (2)
C4—C5—H5A	119.7 (13)	N1—C14—H14C	110.9 (15)
C6—C5—H5A	120.0 (13)	H14A—C14—H14C	110 (2)
C7—C6—C5	120.25 (18)	H14B—C14—H14C	104 (2)
C7—C6—H6A	118.6 (13)	C12—C15—H15A	108.7 (13)
C5—C6—H6A	121.1 (13)	C12—C15—H15B	112.9 (14)
C6—C7—C8	120.05 (18)	H15A—C15—H15B	111 (2)
C6—C7—H7A	122.2 (12)	C12—C15—H15C	113.7 (15)
C8—C7—H7A	117.7 (12)	H15A—C15—H15C	104 (2)
C7—C8—C3	119.42 (17)	H15B—C15—H15C	106 (2)
C7—C8—C9	120.71 (16)	C13—C16—H16A	110.0 (14)
C3—C8—C9	119.63 (15)	C13—C16—H16B	112.3 (13)
O3—C9—C8	112.25 (14)	H16A—C16—H16B	111 (2)
O3—C9—C1	111.38 (14)	C13—C16—H16C	112.0 (16)
C8—C9—C1	111.91 (14)	H16A—C16—H16C	106 (2)
O3—C9—C10	105.32 (14)	H16B—C16—H16C	105 (2)
C8—C9—C10	109.17 (14)		
			
C2—N1—C1—O1	172.69 (16)	C3—C8—C9—C1	−29.7 (2)
C14—N1—C1—O1	−9.2 (3)	C7—C8—C9—C10	−86.56 (19)
C2—N1—C1—C9	−12.7 (2)	C3—C8—C9—C10	87.76 (19)
C14—N1—C1—C9	165.40 (16)	O1—C1—C9—O3	−27.0 (2)
C1—N1—C2—O2	172.40 (17)	N1—C1—C9—O3	158.35 (15)
C14—N1—C2—O2	−5.6 (3)	O1—C1—C9—C8	−153.60 (16)
C1—N1—C2—C3	−10.5 (2)	N1—C1—C9—C8	31.8 (2)
C14—N1—C2—C3	171.54 (16)	O1—C1—C9—C10	87.24 (19)
O2—C2—C3—C8	−170.30 (17)	N1—C1—C9—C10	−87.39 (18)
N1—C2—C3—C8	12.7 (2)	O3—C9—C10—C11	−179.26 (15)
O2—C2—C3—C4	11.0 (3)	C8—C9—C10—C11	−58.5 (2)
N1—C2—C3—C4	−166.05 (16)	C1—C9—C10—C11	62.40 (19)
C8—C3—C4—C5	−0.6 (3)	C13—O4—C11—C12	−0.42 (18)
C2—C3—C4—C5	178.13 (17)	C13—O4—C11—C10	177.85 (15)
C3—C4—C5—C6	−0.8 (3)	C9—C10—C11—C12	95.1 (3)
C4—C5—C6—C7	1.1 (3)	C9—C10—C11—O4	−82.6 (2)
C5—C6—C7—C8	−0.1 (3)	O4—C11—C12—N2	0.00 (19)
C6—C7—C8—C3	−1.3 (3)	C10—C11—C12—N2	−177.81 (19)
C6—C7—C8—C9	173.05 (17)	O4—C11—C12—C15	178.81 (18)
C4—C3—C8—C7	1.6 (3)	C10—C11—C12—C15	1.0 (4)
C2—C3—C8—C7	−177.07 (16)	C13—N2—C12—C11	0.4 (2)
C4—C3—C8—C9	−172.78 (16)	C13—N2—C12—C15	−178.48 (17)
C2—C3—C8—C9	8.5 (3)	C12—N2—C13—O4	−0.7 (2)
C7—C8—C9—O3	29.8 (2)	C12—N2—C13—C16	177.28 (19)
C3—C8—C9—O3	−155.84 (15)	C11—O4—C13—N2	0.75 (19)
C7—C8—C9—C1	155.93 (16)	C11—O4—C13—C16	−177.54 (16)

### Hydrogen-bond geometry (Å, °)

**Table:**

*D*—H···*A*	*D*—H	H···*A*	*D*···*A*	*D*—H···*A*
O3—H1O3···N2^i^	0.87 (3)	2.04 (3)	2.847 (2)	153 (3)
C16—H16A···O1^ii^	0.96 (3)	2.28 (3)	3.162 (2)	153 (2)
C16—H16B···O1^iii^	1.01 (3)	2.50 (3)	3.270 (2)	132.9 (19)

Symmetry codes: (i) *x*+1, *y*, *z*; (ii) *x*−1, *y*, *z*; (iii) −*x*+2, −*y*+1, −*z*+1.

## References

[bb1] Allen, F. H., Kennard, O., Watson, D. G., Brammer, L., Orpen, A. G. & Taylor, R. (1987). *J. Chem. Soc. Perkin Trans. 2*, pp. S1–19.

[bb2] Bernstein, J., Davis, R. E., Shimoni, L. & Chang, N.-L. (1995). *Angew. Chem. Int. Ed. Engl.* **34**, 1555–1573.

[bb3] Bruker (2009). *APEX2*, *SAINT* and *SADABS* Bruker AXS Inc., Madison, Wisconsin, USA.

[bb4] Chen, Y.-H., Zhang, Y.-H., Zhang, H.-J., Liu, D.-Z., Gu, M., Li, J.-Y., Wu, F., Zhu, X.-Z., Li, J. & Nan, F.-J. (2006). *J. Med. Chem.* **49**, 1613–1623. 10.1021/jm050896o 16509578

[bb5] Cosier, J. & Glazer, A. M. (1986). *J. Appl. Cryst.* **19**, 105–107.

[bb6] Cremer, D. & Pople, J. A. (1975). *J. Am. Chem. Soc.* **97**, 1354–1358.

[bb7] Hall, I. H., Chapman, J. M. & Wong, O. T. (1994). *Anticancer Drugs*, **5**, 75–82. 10.1097/00001813-199402000-00012 8186434

[bb8] Malamas, M. S. & Hohman, T. C. (1994). *J. Med. Chem.* **37**, 2043–2058. 10.1021/jm00039a017 8027986

[bb9] Mitchell, G., Clarke, E. D., Ridley, S. M., Bartlett, D. W., Gillen, K. J., Vohra, S. K., Greenhow, D. T., Ormrod, J. C. & Wardman, P. (2000). *Pest. Manag. Sci.* **56**, 120–126.

[bb10] Mitchell, G., Clarke, E. D., Ridley, S. M., Greenhow, D. T., Gillen, K. J., Vohra, S. K. & Wardman, P. (1995). *Pestic. Sci.* **44**, 49–58.

[bb12] Sheldrick, G. M. (2008). *Acta Cryst.* A**64**, 112–122. 10.1107/S0108767307043930 18156677

[bb13] Spek, A. L. (2009). *Acta Cryst.* D**65**, 148–155. 10.1107/S090744490804362X PMC2631630 19171970

[bb11] Subbiah Pandi, A., Rajakannan, V., Velmurugan, D., Parvez, M., Kim, M.-J., Senthilvelan, A. & Narasinga Rao, S. (2002). *Acta Cryst.* C**58**, o164–o167. 10.1107/s0108270102000896 11870315

[bb14] Wang, X.-L., Tian, J.-Z., Ling, K.-Q. & Xu, J.-H. (2000). *Res. Chem. Intermed.* **26**, 679–689.

